# New Risk Situations Related to Low Noise from Electric Vehicles: Perception of Workers as Pedestrians and Other Vehicle Drivers

**DOI:** 10.3390/ijerph17186701

**Published:** 2020-09-14

**Authors:** María Carmen Pardo-Ferreira, Juan Antonio Torrecilla-García, Carlos de las Heras-Rosas, Juan Carlos Rubio-Romero

**Affiliations:** School of Industrial Engineering, Universidad de Málaga, 29071 Málaga, Spain; juantorrecilla@uma.es (J.A.T.-G.); chr@uma.es (C.d.l.H.-R.); juro@uma.es (J.C.R.-R.)

**Keywords:** electric vehicles, risk perception, pedestrian, road users, road traffic safety, low noise, occupational health and safety

## Abstract

Sales of electric and hybrid electric vehicles are increasing steadily worldwide, and consequently their presence increases in city areas. At low speeds, the low levels of noise produced by these vehicles could become a new risk factor for road users. However, the magnitude of the risk has not been accurately determined. In addition, its inclusion in the work environment could pose a new risk that should be managed. Thus, in relation to low noise levels of electric and hybrid vehicles, this study aimed to characterise the risk situations and determine the risk perception of workers as pedestrians and internal combustion engine vehicle drivers coming into contact with these vehicles. The data were extracted from 417 questionnaires filled out by the employees of public service companies who come into contact with electric and hybrid vehicles during their working day in the city of Málaga, in the region of Andalusia, Spain. According to the experiences reported, it seems that the risk due to the low noise levels of electric vehicles is moderate and does not reach alarming levels. These risk situations usually occurred in low speed urban areas, particularly when crossing the road, or in semi-pedestrian areas. Almost half the respondents considered that the electric vehicle poses a risk to other road users because it is more difficult to hear, and they believe it likely that other road users could be injured. Despite that risk, pedestrians did not change their way of walking or moving around the parking areas and other areas of the company. Electric and hybrid electric cars are now required to produce sound when travelling at low speeds. Nevertheless, the effectiveness of this measure should be assessed once implemented and future research should explore alternative non-acoustic measures.

## 1. Introduction

The number of electric cars registered reached an all-time high in 2017, with more than 1 million units sold worldwide. In fact, the global stock of electric cars surpassed 3 million vehicles in 2017, after passing the threshold of 1 million in 2015 [1]. By 2019, that number had swelled to 7.2 million [2]. Forecasts indicate that the number of electric vehicles (EVs) will continue to increase in the future and will eventually replace internal combustion engine (ICE) vehicles in cities. EVs are characterised by being environmentally friendly and silent. Electric engines have several advantages over conventional combustion engines, such as greater efficiency, durability, lower maintenance costs, and lower noise levels [3]. Nevertheless, this latter characteristic has generated much controversy in recent years [4,5,6,7].

In general, road traffic noise in urban environments is generated by vehicles and is mainly due to the friction of the tires and the noise of the engine. At speeds of over 30 km/h, the noise of tire friction overcomes the noise of the engine [8]. However, at low speeds, the predominant noise is that of the engine. Specifically, EVs and hybrid electric vehicles (HEVs), which operate in electric mode at low speeds, have quieter engines at these speeds. This, coupled with the increased use of low noise road surfaces, could pose a threat to the safety of vulnerable road users [9] who rely on their senses of sight and hearing to navigate road traffic safely in urban environments, since the paucity of auditory cues associated with the approach of these vehicles at low speeds increases the risk of pedestrian accidents [10,11,12]. Recent work has focused on analysing the relationship between the age of younger pedestrians and the ability to correctly collect and interpret visual and auditory signals from road traffic [13,14], which indicates that in children under 11 years of age the interpretation of auditory signals can be erroneous. To this aspect, it should be added that the increasing use of portable audiovisual aids while walking, such as cell phones, headphones, tablets, etc., is making it difficult for mainly young people to perceive vehicles.

The absence of acoustic signals that help detect the presence of an EV or HEV can compromise pedestrian safety, especially in low visibility areas or in the case of blind people. According to Morgan et al. [15], the scale of the problem is currently very small, and Cocron and Krems [16] suggested that dangers associated with low noise emissions might be less significant than previously expected. Nevertheless, the magnitude of the risk is still not known accurately, although it seems clear that the low noise levels typical of EVs and HEVs at low speeds presents a new risk to road users.

Vehicles and individuals live together simultaneously in our cities. Traffic regulations, road design, pedestrian walkways and other elements form a shared environment where the pedestrian and the vehicle inevitably have to use the same space. The pedestrian is a very important actor in this situation, if the sound of vehicles moving faster than pedestrians seems important to us, so is the behaviour and sensory capabilities demonstrated by individuals. The responsibility is not unique to drivers of vehicles that do not emit sound, pedestrians also exercise personal responsibility. The increasing use of headsets, cell phones and other portable devices is present in a large part of today’s accidents, regardless of whether a VE or HVE was involved. Human beings have used their senses to prevent and avoid dangers since prehistoric times. The current advance in technology, which allows us to move around in increasingly silent vehicles, could be used to improve our environment, which does not imply that drivers and pedestrians have to adapt to this new situation.

Having identified this issue, the question arises of whether the absence of noise can contribute to the increase in the number of crashes involving EVs and HEVs and pedestrians or other road users. Some countries have performed statistical analyses of vehicle crashes involving pedestrians, comparing HEVs and ICE vehicles. The NHTSA (National Highway Traffic Safety Administration), in their study on the Incidence of Pedestrian and Bicyclist Crashes by Hybrid Electric Passenger Vehicles [17], found that HEVs have a higher incidence of pedestrian and bicyclist crashes than ICE vehicles in certain vehicle manoeuvres at low speeds [17]. Likewise, a study conducted by the UK Transport Research Laboratory suggests that proportionately more EVs and HEVs hit pedestrians than ICE vehicles [15]. However, this study highlighted three notable limitations on the validity of the results: the lack of information on usage patterns and total mileages; the small size of the database; the impossibility of determining whether HEVs were operating in full electric mode at the time of the crash. For these reasons, it cannot be determined whether the lack of noise from EVs and HEVs was a contributory factor in the crash [15]. As a result, the NHTSA study has been widely criticised [7,15]. Studies performed in other countries, such as Netherlands [18] and the Japan [19], did not find a higher incidence of pedestrian crashes of HEVs with respect to ICE vehicles, and to date there has been little direct evidence to conclusively indicate that low noise levels cause more crashes.

Researchers evaluating the risks associated with low noise emissions from EVs and HEVs have used various different approaches. According to Misdariis and Cera [20], three types of studies can be established. First, those that analyse the impact of EVs and ICE in terms of noise levels from the perspective of both pedestrians and drivers. Specifically, studies focussing on pedestrians mainly explored auditory detectability [11,21,22,23,24,25,26,27], and those that focus on drivers mainly analyse their perception of risk [16,28,29,30]. Second, studies analysing the extent to which low noise levels emitted by the EVs [31,32,33] can influence crashes. Third, studies focussing on potential solutions for the issue of low noise emissions of EVs. Proposals can be generally grouped under acoustic solutions, namely, adding noise to the EV [4,5,7,34,35,36], and non-acoustic solutions [5,7].

Non-acoustic solutions could be used to reduce risk without losing the benefit of reduced acoustic pollution in cities. Although this approach has several benefits to human health, it has not been widely researched. Governments are loath to add noise to these vehicles, and accordingly have developed different regulations that require all silent vehicles to incorporate Acoustic Vehicle Alerting Systems (AVAS). The law comes into force from 2019 in Europe [37], and in 2020 in the United States [38] (National Highway Traffic Safety Administration, 2018). However, Sandberg [7] points out that this solution can have the opposite effect and lead drivers to transfer the responsibility of avoiding collisions to pedestrians, on the assumption that since their vehicles are equipped with AVAS, it is the pedestrian’s responsibility to hear them and adequately evaluate the risk. Cocron and Krems [16], meanwhile, point out that technological solutions alone might not solve the problem, and found that drivers adapted their driving behaviour to compensate for low noise emissions. According to Hoogeveen [29], when the first cars were introduced in cities, pedestrians changed their behaviour and paid more attention when crossing the road, suggesting that pedestrians may be able to adapt their behaviour over time. However, the small number of electric vehicles in traffic that exists today and the short exposure time of pedestrians to these vehicles do not allow this suggestion to be verified yet.

No studies have, as yet, analysed the risk situations and the perceived risk from the point of view of the pedestrian or ICE vehicle drivers. All existing studies use auditory detectability, statistical analysis, or other tests, but the individual perception of pedestrians has not been examined. This study is intended to characterise risk situations, and to determine the risk perception of the low noise levels of EVs and HEVs held by workers in contact with these vehicles as pedestrians and ICE vehicle drivers. Although the number of electric vehicles is gradually increasing in cities, there are still very few compared to ICE vehicles. Therefore, in order to ensure that pedestrians and ICE vehicle drivers studied are in frequent contact with EVs and HEVs, the study focuses on an occupational field in which workers from certain companies have had contact with EVs and HEVs in their work environment for at least two years. This selection, applied in the search for respondents, could be interpreted as a bias in the research. The reason for conducting surveys of people who meet the profile described above is generated by the difficulty of finding citizens in the total population who have had or have contact with this type of vehicle, since the percentage of vehicles of this type is still low, coupled with the importance that this type of risk presents in the workplace. This research is part of a larger project in which the risks and situations caused by the low noise levels of EVs and HEVs have been analysed from the point of view of drivers, pedestrians and experts.

## 2. Materials and Methods

The study was performed in the city of Málaga, which is located in Spain’s Andalusia region. Málaga, on the Mediterranean Costa del Sol, is the southernmost large city in Europe. With a total area of over 395 km^2^ and a population of 570,006 inhabitants in the 2017 census, it is the sixth most populated city in Spain and the second largest in Andalusia. In demographic terms, therefore, it is larger than cities such as Lisbon, Dublin or Manchester.

Hereinafter, the term “electric vehicles” includes electric vehicles and hybrid electric vehicles operating in electric mode at low speeds. Likewise, in the present study, “risk situation” was defined as a situation in which the worker perceives a danger that could damage the health of the people exposed to it or cause material damage. Therefore, a particular and extreme case of risk situations is crashes with EVs or HEVs, since it is a risk situation in which the danger materialised causing an accident. However, due to their importance, when the workers were asked if they had experienced risky situations, they were also asked to specify if these risk situations had led to a crash.

It is important to note that this study is part of a larger project in which the perception of drivers, pedestrians and experts was analysed with a common approach. Initially, the risk perception of drivers regarding the lack of noise was analysed [39]. Next, the risk perception of pedestrians was analysed, and the results are presented in this manuscript. Finally, a panel of experts was carried out to know their perception of risk and analyse the results obtained. For all this, the model proposed by Rundmo and Iversen [40] on risk perception was used as a framework. This model considers that the perception of risk can be divided into two components, the rational component and the affective component. The rational or cognitive component is based on probability judgments and beliefs about traffic risks and the affective component or emotion-based component is based on worry and emotional reactions when thinking of traffic hazards. In addition, studies developed by Crocron and Krems [16] and Labeye et al. [30], which specifically focussing on the risk perception of low noise of electric vehicles, were used as a reference for the development of the questionnaires. Following their investigations, additional aspects were included in the questionnaire items.

**Figure 1:**
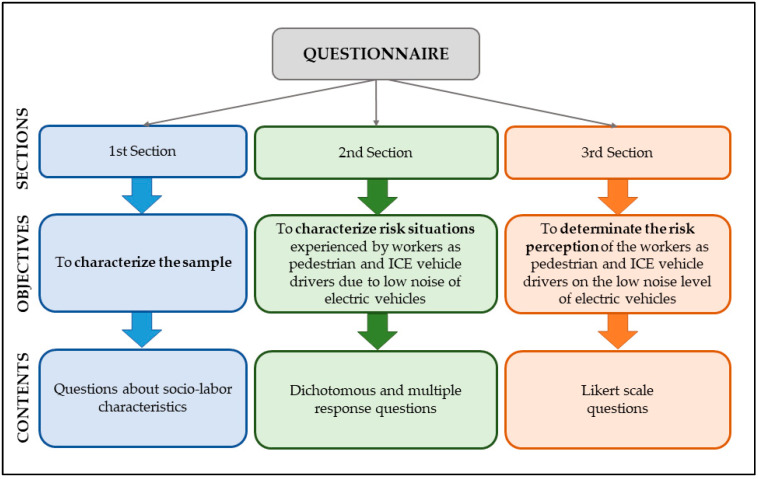
Structure of the questionnaire for workers as pedestrians and internal combustion engine (ICE) vehicle drivers about the situations and risk related to low noise levels of electric vehicles (EVs) and hybrid electric vehicles (HEVs).

In line with this, to evaluate the risk situations experienced by workers as pedestrians and ICE vehicle drivers, an ad hoc questionnaire was designed based on the studies of Rundmo and Iversen [40], Cocron and Krems [16], Labeye et al. [30] and Pardo-Ferreira et al. [39]. The questionnaire was divided into three sections, as shown in Figure 1. Initially, the workers who participated in the study were given an introduction to the research project and the aim of the questionnaire in order to ensure they understood the context of the research in which they were participating. The questionnaire also included a comment box in which they could express opinions, observations and additional comments on the study topic.

The first section was very brief and collected information about the social and employment status of the participants with the aim of characterising the sample. The second section was intended to identify and characterise the risk situations caused by the low sound levels of EVs experienced by study participants as workers. This section was the most extensive and included some dichotomous and some multiple response questions. In this way, the questions directly focused on the characteristics and the specific conditions in which the risk situations occurred, allowing participants to select more than one response if necessary.

The third questionnaire section analysed the risk perception of pedestrians and other vehicle users of the low noise levels of electric vehicles. Following the same structure of the model developed by Labeye et al. [30], and with the aim of making future comparisons, the section was composed of 6 items rated on a 6-point Likert scale from 1 “very strongly disagree” to 6 “very strongly agree”. These items were adapted from the study by Pardo-Ferreira et al. [39], in turn based on earlier studies [16,30,40], which focused on the risk perception of EV drivers on the low noise emissions of EVs. One item was selected for each aspect of the risk perception evaluated, such as low noise level, safety, comfort, cognitive and affective component. A final item was included to determine the pedestrians’ opinions on the possibility of implementing an idling noise in EVs. Pedestrians and ICE vehicle drivers were also asked to assess the risk level of situations that could be caused by the low noise emission of electric vehicles. The respondents had to rate the risk level from 1 “not dangerous at all” to 10 “very dangerous”.

**Figure 2:**
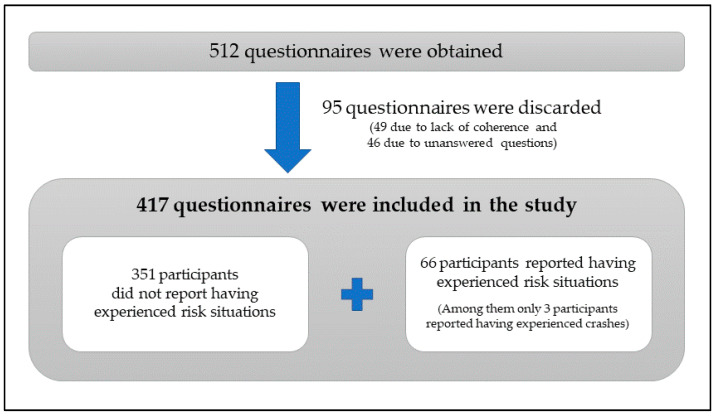
Analysis process applied to the questionnaires.

The participants completed the questionnaire at their workplaces. In some cases, the questionnaire was sent to them by email so that they could answer it online. In other cases, workers did not have computers at their workplace and shift changes were used for workers to complete the paper questionnaire. Figure 2 presents a summary of the questionnaire analysis process. Initially, 512 questionnaires were obtained, of which 95 were discarded due to lack of coherence or predominance of unanswered questions. Finally, 417 questionnaires were obtained for analysis. The collected information was analysed in the results section. Among the 417 participants there were 66 participants who reported having experienced risk situations. Due to their special interest, their responses are analysed separately and presented in the results section.

### Socio-Demographic Characteristics of the Participants

The main characteristics of the study participants are shown in Table 1. All worked in public service companies in Málaga, had an average age of 42 years, and included pedestrians and ICE vehicle drivers. The companies were responsible for public services in the city, such as water supply, environment, operational services, solid waste collection, or street cleaning, and all included EVs and HEVs in their fleet. The vehicles are driven, usually at low speeds, through the facilities and parking areas of the companies used by pedestrians and ICE vehicle drivers. Only 5% of the workers polled had held their job for less than two years. Furthermore, the majority of participants worked as operators, and as such frequently moved around within the companies’ facilities and also provided services outside, that is, in the city. Consequently, most of them have been in contact with EVs and HEVs during their working day for 2 years or more, since the EVs and HEVs were acquired by companies between 2 and 4 years before carrying out the present study. This concentration in space and time of EVs and HEVs and pedestrians and ICE vehicle drivers provided a natural laboratory environment for this exploratory study.

## 3. Results

### 3.1. Characteristics of Risk Situations

**Figure 3:**
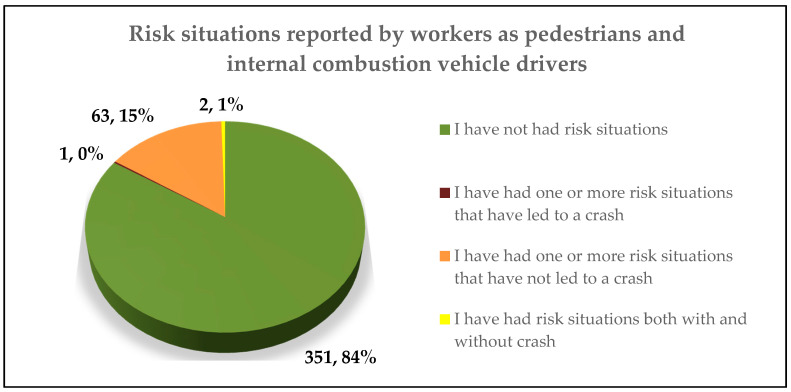
Results of risk situations reported by workers (n = 417).

In order to identify risk situations related to EVs and HEVs, the workers were asked if they had experienced a risk situation due to the low level of noise of EVs and HEVs. Furthermore, they were asked to differentiate whether these risk situations had led to a crash or not. Figure 3 shows the responses obtained from the 417 participants.

According to this figure, 66 participants reported having experienced risk situations. The responses of these 66 participants (15.8% of total participants) were analysed separately in order to perform an in-depth analysis and characterise the reported events. The results of this analysis are summarised in Table 2.

The participants reported a low frequency of crashes with EVs or HEVs during the time they had been in contact with these vehicles in the work environment, that is, between two and four years. Almost two thirds of respondents who reported experiencing risk situations indicated that these situations occurred once or several times per year.

Questions on the types of risk situations were multi-response, so many pedestrians and ICE vehicle drivers selected more than one answer. When these events occurred, more than half of the participants were pedestrians. Analysing the type of vehicles involved showed that both electric cars and hybrid cars were involved in more than half of the risk situations reported. In fact, the three participants who had experienced a crash indicated that the type of vehicle involved was an EV or HEV. The results also show that slightly more than a quarter of the participants indicate that other types of EVs and HEVs were involved in these situations. The reason is that one of the companies collaborating in this study has other types of small EVs that travel daily through the city for waste management services and street cleaning. These are Piaggio Porter Rossi waste collection vehicles with containers. Finally, regarding hybrid electric cars, many of the situations involved hybrid taxis, which, as the participants observed, are increasingly prevalent in the city of Málaga.

Regarding the areas or settings in which the risk situations occurred, the majority of participants reported having experienced them in the urban setting. This may be due to the fact that these workers spend a large part of their working day on the streets of the city, since they work in public service companies. In the comment box, some participants highlight the problem of hearing EVs and HEVs in these areas *“I did not see the car until it was 3 m away”*. Especially when crossing the road, *“I did not hear him coming and I thought it was safe to cross”, “when crossing a street, I did not notice the vehicle because I did not hear it”* or in semi-pedestrian areas, *“in pedestrian streets, you cannot hear the vehicle” “the danger may lie in pedestrian streets”.*

Problems in parking areas were also reported by some participants. Accordingly, observations such as “in zones shared by vehicles and pedestrians, such as parking lots, in which pedestrians do not perceive the presence of an electric vehicle” were recorded. Some of these events occurred within the company’s facilities, such as “I did not notice that the electric car was starting to circulate in the indoor parking lot” or “I was walking along the track and I did not hear the car”.

### 3.2. Risk Perception of Pedestrians and ICE Vehicle Drivers

**Figure 4:**
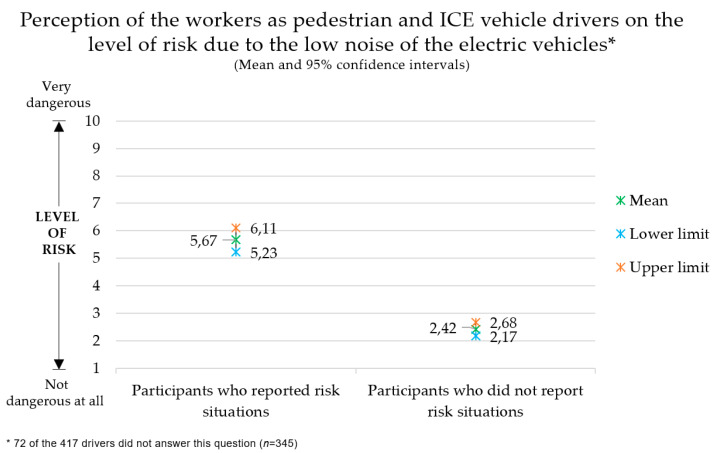
Level of risk of situations that could be caused by the low noise emissions of EVs, assessed by workers as pedestrians and internal combustion engine vehicle drivers (n = 346).

All 417 workers surveyed, both pedestrians and ICE vehicle drivers, were asked to rate the level of risk related to the low level of noise of EVs and HEVs to other road users (pedestrians, cyclists, drivers of other vehicles) on a scale of 1 to 10. Figure 4 shows the results obtained. The average level of risk perceived by all participants was 3.03 (SD = 2.46). In fact, almost 41% of respondents described the risk level of situations involving EVs and HEVs as very low (1).

A statistical analysis was performed to find significant differences in the perceived risk of participants who reported risk situations and those who did not. The Kolmogorov–Smirnov test showed that the data were not normally distributed, so the non-parametric Mann–Whitney test was applied. The results showed a significant difference in the level of risk perceived by participants who reported experiencing risk situations and those who did not, U = 2444.500, Z = −9.708, p = 0.000. In fact, those who were involved in these events rated the risk higher.

In the case of workers who reported having experienced a risk situation (n = 66), a descriptive data analysis was used to explore the impact of the different characteristics of involved risk situations on risk perception. According to this, the significance of the difference in the types of vehicle involved, how the worker got around, the circumstances in which it occurred or the area in which it occurred, on risk perception was tested using one-way ANOVA and Tukey’s post-hoc tests. These variables were previously confirmed to be normal using the Shapiro–Wilk test and the equality of variance was verified using the Levene test. The results indicate that there are no significant differences between the level of perceived risk according to the characteristics of the risk situations experienced by the workers, since in all cases it was fulfilled that p > 0.05 as shown in the Table 3.

Participants were also asked to rate their agreement with six statements on a 6-point Likert scale. The results obtained are shown in Appendix A. Following the example of Labeye et al. [30], the percentage of “agreement” and the margin of error for each item were included. This percentage was calculated from the aggregate of the three upper values of the Likert scale (4-5-6).

Initially, the perception of the effect of electric vehicles on road safety was studied. The results indicated that more than half of the participants considered that electric vehicles posed a greater risk to other road users because they are more difficult to hear. In this respect, one participant indicated, *“it is fundamental for pedestrians to be able to detect the presence of vehicles, and the absence of noise is dangerous for pedestrians”.*

Analysing the perceived comfort, the majority of participants considered that the silence of the electric vehicles was pleasant. The cognitive component was evaluated, considering the probability that electric vehicle drivers could injure or damage other road users. The majority of participants disagreed with the statement that it is very improbable that this would occur. Therefore, they believe that it is probable that other users could be injured or damaged.

Following this, the influence of the silence of electric vehicles on the behaviour of pedestrians and other users was analysed. The findings showed that most had not changed their way of walking or moving around in the parking areas, garages and other areas of the company due to the lack of noise of the electric vehicles.

Finally, an item was included in the survey to estimate the possibility of incorporating an additional sound to these vehicles when the engine is in idle. In this case, the distribution of the answers presents some symmetry, without obtaining a result significantly for or against incorporating engine idling sounds. In fact, the percentage in favour of the incorporation of noise to the engine at idle was 50% with the average of 3.55 (SD = 1.8).

### 3.3. Additional Comments from Pedestrians and ICE Vehicle Drivers

As mentioned above, participants were allowed to write additional comments at the end of the questionnaire in the comment box. Some pedestrians and users of ICE vehicles argued in favour of EVs and HEVs for different reasons, such as the positive impact of these vehicles on the environment. Thus, some of them commented *“I think electric vehicles are great, because they help with the pollution of the planet and also do not pollute acoustically”* or *“Electric vehicles are quieter and pollute less, making them better”*. Some participants also recognised potential problems but considered that they could be solved by extreme caution, and the environmental advantage was reiterated: *“Undoubtedly, it is obvious that the low noise level can have a certain impact on both pedestrians and other types of vehicles. However, it is imperative to use extreme caution when driving these vehicles and when walking along the road, in order to avoid any type of crash. I think that electric vehicles contribute to better environmental conservation”.*

Others argued that it was important to keep an eye out for possible risk situations: “I have not had a crash, nor have I witnessed any crashes caused by an electric vehicle due to the low noise level. In addition, one of the most important senses for a pedestrian or driver is sight, and most modern vehicles are relatively soundproof”.

Some participants pointed out the importance of all road users, both drivers and pedestrians, respecting road safety rules, whether or not EVs are involved: *“As long as the people follow road safety rules, there should be no crashes, and the elimination of acoustic contamination is possible”* or *“The noise does not imply that the driver does not respect traffic regulations. Pedestrians must also respect them”*.

On the other hand, some participants considered that possible risk situations have been the responsibility of the driver and not caused by the absence of noise: *“In my opinion, it is not the vehicle, it is the driver that drives it”* or *“The important thing is not that the car makes a noise when driving. The interesting thing is that the driver is aware of this situation and drives properly, paying special attention in pedestrian zones, parking, etc.”.*

Although some participants were strongly in favour of the absence of noise, some highlighted the danger related to the lack of noise: *“In general, I understand that noise is a risk for pedestrians” or “I have not had any risk situation, but when I walk I do not hear them, and that is a problem for someone working in the street”.* So, some participants considered it necessary to take measures, such as *“they should make some noise to see them coming”* or adding *“to the car some kind of sound or a type of safety braking in case the driver is distracted”.*

## 4. Discussion

The results show that only three of the 417 participants reported crashes involving EVs on the road or in their company’s facilities. Regarding the level of risk perceived by pedestrians and ICE vehicle drivers, the results initially seem to indicate a relatively low level of risk. However, an in-depth analysis indicated a significant difference in the assessment of the level of risk between the two groups of participants. Thus, participants who had experienced risk situations reported a higher level of risk than participants who declared they had not experienced any such situation. Nevertheless, only 15.8% of the participants reported having experienced risk situations, which could explain the average value obtained. In addition, this finding also suggests that past experience with risk situations correlates with the level of perceived risk for pedestrians and ICE vehicle drivers due to the EV’s lack of noise. 

Admittedly, it is mandatory to add sound to EVs and HEVs manufactured from July 2019, and this will be mandatory for any EVs or HEVs on the roads from 2021 in Europe. In the United States, this measure has been postponed until September 2020 to clarify the technical requirements for these vehicles. Nevertheless, other complementary actions should be undertaken by governments, since the problem is not entirely solved by simply adding sound to these vehicles. Organising campaigns to raise awareness of the dangers of these vehicles and giving this information to learner drivers appear to be the cornerstones of improvement.

In addition, the key objective of adding sound to EVs should not be forgotten, and the effectiveness of this measure should be evaluated when the number of vehicles increases, as it may not be as effective as was originally assumed. Even the efficiency of other non-acoustic technical measures, such as automatic braking systems, could be evaluated. In addition, future research should explore alternative non-acoustic measures and analyse the effectiveness of those that already exist, such as automatic braking systems.

Nevertheless, this difference in the level of risk between people who had experienced risk situations with EVs or HEVs and those who had not, was not found in a previous study carried out in the present project and focused on workers who drive EVs and HEVs [39]. Beyond this, interestingly, the level of risk generally reported was higher in the case of EV drivers [39] than in the case of pedestrians and ICE vehicle drivers. Furthermore, it should be noted that the percentage of electric vehicle drivers who had experienced risk situations was higher, reaching 62%. This could be caused by the longer uninterrupted exposure of electric vehicle drivers. In this research, it is important to remember that the people surveyed are workers who usually live with EVs or HEVs in their workplace, which may have influenced the moderate risk perception that this group has presented.

Regarding risk perception, the results show that, on the one hand, workers as pedestrians and ICE vehicle drivers considered that electric vehicles posed a higher risk for road users, since it is more difficult to notice the oncoming vehicle. However, they recognised that the silence of electric vehicles is pleasant. Similar results have been reported in several studies focussing on the risk perception of drivers [16,29,30]. On the other hand, the majority of respondents indicated that it is likely that pedestrians could be injured by electric vehicle because of the low noise level. Despite this, most acknowledged that they had not changed their way of moving around the parking areas, garages and other company facilities, neither were they overly concerned that electric vehicle drivers could injure other road users due to low noise level. This transfer of responsibility or blame to the driver was also observed to a certain extent in this study. In this sense, Sandberg [6] indicates that the responsibility of avoiding a crash with a pedestrian traditionally falls on the driver. In addition, Sandberg [6] points out that adding sound to EVs and HEVs may cause the driver to transfer this responsibility to the pedestrian and other drivers, in the belief that they should hear the sound and be warned. As a result, this measure would be counterproductive.

According to Morgan [15], EVs and HEVs are mainly used in urban areas, an observation echoed in our study. With regard to high risk manoeuvres, Hanna [17] notes that crashes involving HEVs and pedestrians usually occur at very low speed, such as when vehicles are turning, slowing or stopping, backing up, or entering or leaving a parking space. Similarly, our results show that the reported risk situations took place at low speeds, particularly when vehicles were driving straight, and pedestrians were crossing the street or walking in parking areas. In addition, our findings reveal that semi-pedestrian zones shared with vehicles and pedestrians, but with pedestrian preference, can be considered special risk areas. These areas are becoming increasingly common in city centres. In these locations, pedestrians are not fully alert to oncoming vehicles and this increases the risk. Nevertheless, the low speed of the vehicles reduces the risk of serious injury.

In the comment box, pedestrians and ICE vehicle drivers expressed some aspects of special interest, such as the positive impact of EVs on the environment, which has already been highlighted as a valued feature of these vehicles [41,42,43] and the predominantly positive attitudes towards EVs [28].

## 5. Limitations

The main limitations of this study are summarised below. Firstly, we had no data on the exposure time of pedestrians and ICE vehicle drivers. While it is known that during their working day they are in the same environment as EVs and HEVs, we do not know how often they encounter these vehicles. It seems likely that there is greater chance of exposure at the beginning and at the end of the working day, given the concentration of workers and vehicles in the same space and time due to shift change.

Secondly, there are limitations related to the sample. All participants work in public service companies in the city of Málaga, as mentioned above. Ideally, the scope of the study should be extended to include other cities and private companies. Widening the range of participants to pedestrians and ICE vehicle drivers not linked to any particular company would further improve the scope of the study. However, it is difficult to secure the participation of individuals frequently exposed to EVs and HEVs outside the corporate setting. Nevertheless, the number of EVs and HEVs is expected to increase significantly in the short term, and this would increase the feasibility of such an in-depth study.

Finally, the results obtained in this study are based on the self-reported perceptions of workers as pedestrians and ICE vehicle drivers, so we cannot rule out the element of subjectivity in their perception of reality and in their opinions. In addition, there is always the risk of recall bias when remembering risk situations with EVs or HEVs. That is, asking participants whether EVs might be dangerous because of low noise might have prompted them to think about situations when they had a near miss with an EV or HEV. However, they may have had similar experiences with ICE vehicles that they have not considered in the same way.

## 6. Conclusions

The environmental benefits and silence of EVs and HEVs have once again been highlighted by workers as pedestrians and ICE vehicle drivers that have participated in this study. In line with the findings of other studies, pedestrians have reported difficulties in detecting the approach of EVs and HEVs, and therefore the risk associated with this danger becomes a reality. However, it seems this risk perception is moderate among workers who habitually coexist with these vehicles during their workday. In fact, the current low level of risk due to the low noise emissions of EVs and HEVs may be directly related to other factors, such as time spent on public roads and facilities, or the market penetration of EVs in urban areas. As expected, these risk situations usually occurred once or several times per year, with involvement of pedestrians and electric cars at low speeds in urban areas. They also highlight the existing risk when crossing the road or in semi-pedestrian areas.

Our findings suggest the need for measures aimed at eliminating or minimizing risk. As possible lines of research to be developed in the future, we understand that it is necessary to study in depth different solutions to minimise the risk due to the absence of noise from EVs and HEVs. Non-acoustic solutions have been ruled out by governments, but other types of measures could be adopted. In any case, it is essential also to analyse in future research how to teach drivers, pedestrians and other roads users to coexist with this new type of vehicle. In this sense, it is important for pedestrians to be aware that they can minimise the risk of being involved in a crash with a vehicle by observing road safety rules and taking precautions. We are facing a new scenario, where the increasing use of EVs and HEVs will compose an environment to which it will be necessary to adapt. Large organisations are including fleets of EVs and HEVs in their vehicle fleet, which also opens up a field of research in relation to the occupational health and safety management. It is important that companies that have already purchased these types of vehicles are aware that such use may involve new risks. These companies must include this new situation in their risk assessments and establish the preventive measures that are necessary to eliminate or reduce them. It is therefore necessary to continue analysing the problem in order to help them do so properly.

## Figures and Tables

**Table 1:** Characteristics of the sample of workers (n = 417).

Socio-Demographic Characteristics of the Sample			
Variable	Response Options	Cases	Percent
Gender	Male	320	77%
	Female	97	23%
Age	Less than 25 years	14	3%
	25 to 34 years	67	16%
	35 to 44 years	152	36%
	45 to 54 years	112	27%
	55 to 64 years	40	10%
	Over 64 years	0	0%
	No response	32	8%
Job post	Manager/CEO	4	1%
	Management Position	8	2%
	Technical Position	19	5%
	Operator	386	93%
Seniority in the company	Less than 1 year	10	2%
	More than 1 year and less than 2 years	8	2%
	More than 2 year and less than 3 years	5	1%
	More than 3 year and less than 4 years	6	1%
	4 years or more	334	80%
	No response	54	13%

**Table 2:** Identification and characterization of risk situations by workers (n = 66).

Characterization of Risk Situations			
Variable	Response Options	Cases	Percentage
How many crashes with an electric vehicle have you been involved in due to the lack of noise of these vehicles?	0	63	95%
	1	2	3%
	2	1	2%
How often have you experienced risk situations?	Never	3	5%
	Once a week	3	5%
	Once a month	3	5%
	More than once a month	14	21%
	Once a year	19	29%
	More than once a year	24	36%
How do you get around?	On foot, as a pedestrian	33	50%
	Driving ICE vehicle	24	36%
	Both	7	11%
	No response	2	3%
With what type of vehicle did you have such risk situations? *	Electric car	27	38%
	Hybrid electric car	13	18%
	Electric motorcycle	9	13%
	Hybrid electric motorcycle	1	1%
	Other electric vehicles	19	27%
	No response	2	3%
Under what circumstances did it occur? *	Less than 30 km/h	29	34%
	Driving straight	14	16%
	While overtaking or driving	8	9%
	When the vehicle started or parked	7	8%
	At a traffic light, turning, or at an intersection	8	9%
	In other manoeuvres	16	19%
	No response	3	4%
In what areas did risk situations occur? *	Urban areas	51	73%
	Garage and repair zones	2	3%
	Parking areas	11	16%
	Other company facilities	5	7%
	No response	1	1%

* Multi-response questions.

**Table 3:** Summary of ANOVA analysis of the significance of the characteristics of risk situations on the perception of risk.

	Sum of Squares	df	Mean Square	F	Sig.
How do you get around?	2.021	2	1.01	0.318	0.729
With what type of vehicle did you have such risk situations?	4.183	5	0.837	0.253	0.936
Under what circumstances did it occur?	21.064	6	3.511	1.166	0.337
In what areas did risk situations occur?	20.989	4	5.247	1.783	0.144

## Appendix A

**Table A1:** Results of workers’ responses to the items related to the perception of risk due to the low noise of EVs and HEVs.

Items	Number/Percentage								
Indicate Your Degree of Agreement with the Following Statements	Very Strongly Disagreed (1)	Strongly Disagree (2)	Mostly Disagree (3)	Mostly Agree (4)	Strongly Agree (5)	Very Strongly Agree (6)	No Response	Percentage of Agreement/Margin of Error	Mean/(Standard Deviation)
1. Even though an electric vehicle is harder to hear, other road users such as pedestrians, other drivers, etc. are not at higher risk	88	54	85	74	40	75	1	45%	3.36
	21%	13%	20%	18%	10%	18%	0%	4.78	(1.74)
2. The quietness of an electric vehicle is pleasing	53	35	53	34	49	186	7	65%	4.34
	13%	8%	13%	8%	12%	45%	2%	4.59	(1.86)
3. It is very improbable that electric vehicle drivers could injure pedestrians in a crash due to low noise	105	49	80	68	37	64	14	41%	3.19
	25%	12%	19%	16%	9%	15%	3%	4.71	(1.76)
4. I had to change my way of walking around the parking areas, garages and other areas of the company due to the lack of noise of the electric vehicle	131	54	76	70	24	43	19	33%	2.83
	31%	13%	18%	17%	6%	10%	5%	4.51	(1.68)
5. I am very concerned that drivers of electric vehicles could injure other road users in a crash due to the low noise level	105	66	68	77	52	46	3	42%	3.10
	25%	16%	16%	18%	12%	11%	1%	4.74	(1.69)
6. I would not mind if my electric vehicle had an idle noise so that others could hear it at any time (while parked with engine on)	81	51	72	71	44	93	5	50%	3.55
	19%	12%	17%	17%	11%	22%	1%	4.80	(1.80)

## References

[1] International Energy Agency (2018). Global EV Outlook 2018. Towards Cross-Modal Electrification.

[2] International Energy Agency (2020). Global EV Outlook 2020. Entering the Decade of Electric Drive.

[3] European Environment Agency Electric Vehicles in Europe Electric Vehicles in Europe. https://op.europa.eu/en/publication-detail/-/publication/1a4a941c-9a8d-11e6-9bca-01aa75ed71a1/language-en.

[4] Lee S.K., Lee S.M., Shin T., Han M. (2017). Objective evaluation of the sound quality of the warning sound of electric vehicles with a consideration of the masking effect: Annoyance and detectability. Int. J. Automot. Technol..

[5] Owen J.M. Quiet vehicle avoidance systems for blind and deaf-blind pedestrians. http://citeseerx.ist.psu.edu/messages/downloadsexceeded.html.

[6] Poveda-Martínez P., Peral-Orts R., Campillo-Davo N., Nescolarde-Selva J., Lloret-Climent M., Ramis-Soriano J. (2017). Study of the effectiveness of electric vehicle warning sounds depending on the urban environment. Appl. Acoust..

[7] Sandberg U. (2012). Adding noise to quiet electric and hybrid vehicles: An electric issue. Acoust. Aust..

[8] Garay-Vega L., Hastings A., Pollard J.K., Zuschlag M., Stearns M.D. (2010). Quieter Cars and the Safety of Blind Pedestrians: Phase I.

[9] Mendonça C., Freitas E., Ferreira J.P., Raimundo I.D., Santos J.A. (2013). Noise abatement and traffic safety: The trade-off of quieter engines and pavements on vehicle detection. Accid. Anal. Prev..

[10] Wogalter M.S., Ornan R.N., Lim R.W., Chipley M.R. (2001). On the risk of quiet vehicles to pedestrians and drivers. Proc. Hum. Factors Ergon. Soc. Annu. Meet.

[11] Maffei L., Masullo M., Sorrentino F., Di Gabriele M. (2013). Preliminary studies on the relation between the audio-visual cues’ perception and the approaching speed of electric vehicles. J. Acoust. Soc. Am..

[12] Stelling-Kończak A., Hagenzieker M., Commandeur J.J.F., Agterberg M.J.H., van Wee B. (2016). Auditory localisation of conventional and electric cars: Laboratory results and implications for cycling safety. Transp. Res. Part F. Traffic Psychol. Behav..

[13] Barton B., Lew R., Kovesdi C., Cottrell N., Ulrich T. (2013). Developmental differences in auditory detection and localization of approaching vehicles. Accid. Anal. Prev..

[14] Whitebread D., Neilson K. (2000). The contribution of visual search strategies to the development of pedestrian skills by 4-11 year-old children. Br. J. Educ. Psychol..

[15] Morgan P.A., Morris L., Muirhead M., Walter L.K., Martin J. (2011). Assessing the perceived safety risk from quiet electric and hybrid vehicles to vision-impaired pedestrians. TRL Publ. Proj. Rep..

[16] Cocron P., Krems J.F. (2013). Driver perceptions of the safety implications of quiet electric vehicles. Accid. Anal. Prev..

[17] NHTSA Pedestrian and Bicyclist Crashes by Hybrid Electric Passenger Vehicles. https://crashstats.nhtsa.dot.gov/Api/Public/ViewPublication/811204.

[18] Verheijen E., Jabben J. (2010). Effect of Electric Cars on Traffic Noise and Safety.

[19] JASIC (2009). A Study on Approach Warning Systems for Hybrid Vehicle in Motor Mode.

[20] Misdariis N., Cera A. (2013). Sound signature of Quiet Vehicles: State of the art and experience feedbacks. INTER-NOISE and NOISE-CON Congress and Conference Proceedings.

[21] Ashmead D.H., Grantham D.W., Maloff E.S., Hornsby B., Nakamura T., Davis T.J., Pampel F., Rushing E.G. (2012). Auditory perception of motor vehicle travel paths. Hum. Factors.

[22] Dudenhöffer K., Hause L. (2012). Sound perception of electric vehicles. ATZ Worldw..

[23] Emerson R.W., Naghshineh K., Hapeman J., Wiener W. (2011). A pilot study of pedestrians with visual impairments detecting traffic gaps and surges containing hybrid vehicles. Transp. Res. Part F. Traffic Psychol. Behav..

[24] Garay-Vega L., Pollard J., Guthy C., Hastings A. (2011). Auditory detectability of hybrid electric vehicles by blind pedestrians. Transp. Res. Rec. J. Transp. Res. Board.

[25] Goodes P., Bai Y.B., Meyer E. (2009). Investigation into the Detection of a Quiet Vehicle by the Blind Community and the Application of an External Noise Emitting System. SAE 2009 Noise and Vibration Conference and Exhibition.

[26] Singh S. (2016). Understanding and Improving Methods for Exterior Sound Quality Evaluation of Hybrid and Electric Vehicles. Ph.D. Thesis.

[27] Walker I., Kennedy J., Martin S., Rice H. (2016). How might people near national roads be affected by traffic noise as electric vehicles increase in number? A laboratory study of subjective evaluations of environmental noise. PLoS ONE.

[28] Cocron P., Bühler F., Neumann I., Franke T., Krems J.F., Schwalm M., Keinath A. (2011). Methods of evaluating electric vehicles from a user’s perspective—The MINI E field trial in Berlin. IET Intell. Transp. Syst..

[29] Hoogeveen L.V.J. (2010). Road Traffic Safety of Silent Electric Vehicles. Master’s Thesis.

[30] Labeye E., Hugot M., Brusque C., Regan M.A. (2016). The electric vehicle: A new driving experience involving specific skills and rules. Transp. Res. Part F. Traffic Psychol. Behav..

[31] Fastl H., Kerber S. (2016). Effects of partial masking for vehicle sounds. INTER-NOISE and NOISE-CON Congress and Conference Proceedings.

[32] Kerber S. (2006). The importance of vehicle exterior noise levels in urban traffic for pedestrian—Vehicle interaction. ATZ Worldw..

[33] Kerber S., Fastl H. (2008). Prediction of perceptibility of vehicle exterior noise in background noise. Fortschr. Akust..

[34] Parizet E., Ellermeier W., Robart R. (2014). Auditory warnings for electric vehicles: Detectability in normal-vision and visually-impaired listeners. Appl. Acoust..

[35] Tabata T., Konet H., Kanuma T. Development of Nissan Approaching Vehicle Sound for Pedestrians. https://www-esv.nhtsa.dot.gov/Proceedings/22/files/22ESV-000097.pdf.

[36] Yamauchi K., Menzel D., Takada M., Nagahata K., Iwamiya S., Fastl H. (2015). Psychoacoustic examination of feasible level of additional warning sound for quiet vehicles. Acoust. Sci. Technol..

[37] European Union Regulation (EU) No 540/2014 of the European Parliament and of the Council of 16 April 2014 on the sound level of motor vehicles and of replacement silencing systems, and amending Directive 2007/46/EC and repealing Directive 70/157/EEC 2014. https://eur-lex.europa.eu/eli/reg/2014/540/oj.

[38] National Highway Traffic Safety Administration Minimum Sound for Hybrid and Electric Vehicles. https://www.federalregister.gov/documents/2018/02/26/2018-03721/federal-motor-vehicle-safety-standard-no-141-minimum-sound-requirements-for-hybrid-and-electric.

[39] Pardo-Ferreira M.C., Rubio-Romero J.C., Galindo-Reyes F.C., Lopez-Arquillos A. (2020). Work-related road safety: The impact of the low noise levels produced by electric vehicles according to experienced drivers. Saf. Sci..

[40] Rundmo T., Iversen H. (2004). Risk perception and driving behaviour among adolescents in two Norwegian counties before and after a traffic safety campaign. Saf. Sci..

[41] Graham-Rowe E., Gardner B., Abraham C., Skippon S., Dittmar H., Hutchins R., Stannard J. (2012). Mainstream consumers driving plug-in battery-electric and plug-in hybrid electric cars: A qualitative analysis of responses and evaluations. Transp. Res. Part Policy Pract..

[42] Peters A., Dütschke E. (2014). How do Consumers perceive electric vehicles? A comparison of german consumer groups. J. Environ. Policy Plan..

[43] Rezvani Z., Jansson J., Bodin J. (2015). Advances in consumer electric vehicle adoption research: A review and research agenda. Transp. Res. Part Transp. Environ..

